# Histological determination of cariously altered collagen after dentin caries excavation with the polymer bur PolyBur P1 in comparison to a conventional bud bur

**DOI:** 10.1186/s13005-019-0205-9

**Published:** 2019-07-15

**Authors:** Jeannine Lohmann, Edgar Schäfer, Till Dammaschke

**Affiliations:** 1 Private Practice, An der Sandkull 108 B, 47445 Moers, Germany; 2 Central Interdisciplinary Ambulance in the School of Dentistry, Albert-Schweitzer-Campus 1, building W 30, 48149 Münster, Germany; 3 Department of Periodontology and Operative Dentistry, Westphalian Wilhelms-University, Albert-Schweitzer-Campus 1, building W 30, 48149 Münster, Germany

**Keywords:** Bud bur, Caries excavation, Collagen, Dentin, PolyBur P1, Polymer bur

## Abstract

**Background:**

To compare the polymer bur PolyBur P1 (P1) with tungsten carbide bud bur H1 SE (H1) in removing cariously altered collagen during dentin caries excavation.

**Methods:**

Fifty extracted teeth were split in the center of a carious lesion. The 100 specimens were randomly divided into 5 groups. Five dentists were asked to excavate 10 teeth each: one half with P1 and the corresponding half with H1. The time needed for caries excavation was measured. Subsequently, histological specimens were produced and analyzed by light-microscope after Mallory-Azan-staining. The thickness of remaining cariously altered collagen was measured (< 1 mm or > 1 mm). The results were statistically evaluated.

**Results:**

The average time to excavate a cavity with P1 was 254 (± 148) sec and 202 (± 129) sec with H1. The difference in times was not statistically significant (*p* > 0.05). In the group P1 in 66.1% of the sections cariously altered collagen remained, whereas 33.9% showed sound collagen. In the group H1 45.7% sections had remaining cariously altered collagen and 54.3% showed sound collagen. The difference between P1 and H1 was statistically significant (*p* = 0.004). In the group P1 the layer of cariously altered collagen was significantly more often thicker than 1 mm than in the group H1 (*p* < 0.05). The variable “type of bur” had a statistically significant influence for the presence of cariously altered collagen (*p* = 0.003).

**Conclusions:**

Conventional H1 bud burs were significantly more effective in removing cariously altered collagen during dentin caries excavation than the polymer bur P1.

## Background

The modern concept of “minimal-invasive dentistry” calls for more conservative elimination of bacterially infected and irreversibly demineralized carious dentin, in order to preserve as much as possible remineralizable dentin and avoid pulp exposure [1]. The search for a less aggressive, comfortable, and conservative caries excavation has led to the development of methods which aim at removal of infected dentin only [2]. By removing just the bacterially infected dentin, it should be possible to arrest further progression of the carious lesion. However, there still exist concerns about where to define this caries removal endpoint, because this is hardly achievable clinically [1, 3].

One criterion to distinguish between remineralizable and non-remineralizable dentin may be the hardness of this tissue [1]. The hardness of healthy dentin is between 51 and 65 Knoop Hardness (KHN), varying by depth [4, 5]. Dentin hardness is significantly higher in superficial than in deep dentin [6]. The Knoop Hardness for active caries (= acute caries) was determined to be 6.7 KHN (range 4.4–11.2 KHN), whereas arrested caries (= chronic caries) was shown to exhibit an average hardness of 39.2 KHN (range 16.0–61.0 KHN) [7]. According to other studies, the hardness of caries affected dentin is 30.7 KHN to 33.9 KHN [8] and of unaffected dentin (beneath the caries lesion) about 60 KHN [9]. Anyway, softening fronts always precede the bacterial invasion front of the lesion towards the pulp [7].

Thus, the idea was to develop a device for dentin caries excavation with a hardness of about 50 KHN, thus laying between the hardness of healthy and cariously infected dentin. A first attempt was made by Horiguchi et al. [10]. They developed polycarbonate particles as an abrasive medium from a powder jet device. Polycarbonate particles have a degree of hardness of 40 to 50 KHN, which means that they lie between carious and healthy dentin and can therefore selectively remove infected dentin. Other abrasive media were harder and therefore, did not have this property [10].

Based on these observations, polymer-based bud burs were developed [11]. The first polymer bud bur was launched in 2003 under the brand name SmartPrep (SSWhite, Lakewood, NJ, USA). The paddle-shaped bur has a unique flute design, and is constructed from a medical-grade polyether-ketone-ketone (PEKK) [12]. According to manufacturer’s information, this special polymer composite has a hardness of 50 KHN, which lay therefore between that of healthy dentin and that of cariously infected dentin. (In comparison: a conventional carbide bud bur has 1,600 KHN.) Hence, the cutting edges of the polymer bur wear out in contact with the harder, healthy dentin, so that only cariously infected dentin is selectively excavated in a minimally invasive treatment. As recommended by the manufacturer, the removal of carious dentin starts from the center of the lesion and continues to the periphery. Excavation using polymer burs still requires conventional metal burs to gain access to the lesion and to finish the preparation margins. Excavation is stopped when the instrument becomes macroscopically abraded blunt, and the polymer bur is no longer able to remove dentin. The SmartPrep is intended to be used in particular for excavations close to the pulp to prevent pulp exposure. This polymer bur is used in a lower speed range of 500 rpm to 800 rpm [12–14].

Later, a new and improved polymer bur with the same principle, but reinforced blades, was launched on the market (SmartBur; SSWhite, Lakewood, NJ, USA) [2, 15, 16]. Interestingly, the hardness of the SmartBur was determined to be only 26.6 (± 1.2) KHN [2], which is less than specified by the manufacturer.

The third generation of polymer instruments was released in 2010 (SmartBur II; SSWhiteBurs, Lakewood, NJ, USA) [17–20] and 2011 (PolyBur P1; Komet, Gebr. Brasseler, Lemgo, Germany) [21]. Whereas SmartBur II has a similar cutting edge compared to its predecessor, but a higher hardness, the PolyBur P1 is a new development.

According to the manufacturer’s information PolyBur P1 is indicated for the excavation of soft, cariously infected dentin close to the pulp in clinically asymptomatic deciduous and permanent teeth. The cutting edge of the PolyBur P1 is similar to that of a conventional bud bur. Furthermore, the shank is more delicate, whereby it is suitable for small cavities. Over-excavation by the PolyBur P1 should be avoided due to the hardness of the material. The application is always additional in the area close to the pulp. Contraindications for the use of PolyBur P1 are dark discolored dentin (Maillard reaction), hard but remineralizable dentin, and caries along the enamel-dentin junction. During excavation, the contact pressure must be observed. The elasticity of the shaft is a control function; if the contact pressure is too high, it bends. According to the manufacturer the end point of the excavation is reached when dentin can no longer be removed by the PolyBur P1. The cutting edges do not have to be rounded, this only occurs when working on dentin which is too hard. The range of application is from 2,000 rpm - 8,000 rpm [21].

While SmartPrep [12–14, 22, 23], SmartBur [2, 15, 16, 24, 25], and SmartBurs II [17–20] were sufficiently investigated concerning dentin caries excavation, to the best of the authors´ knowledge there has been only one study assessing the PolyBur P1 so far [21], which, however, only compared PolyBur P1 with SmartBurs II, without any conventional carbide bud burs as a control group. Thus, the aim of the present study was to compare PolyBur P1 with tungsten carbide bud burs in dentin caries excavation in vitro.

The null hypothesis tested in this study was that the polymer bur PolyBur P1 is as effective as a tungsten carbide bud bur (H1 SE; Komet, Gebr. Brasseler, Lemgo, Germany) in removing cariously altered collagen during dentin caries excavation.

## Materials and methods

### Sample collection

For this in vitro study, 50 extracted human carious teeth (anterior, premolars, and molar teeth) without restorations were selected. The exposed carious lesions were occlusal or cervical in the dentin. Immediately after extraction, the teeth were stored in a phosphate-buffered saline solution (PBS solution; pH 7.2) and then kept in the refrigerator at 5 °C for no more than 1 week after extraction.

Using a diamond saw with permanent water cooling (Model 1600, Leitz, Wetzlar, Germany) the teeth were split lengthwise in the center of the carious lesion, creating two corresponding tooth halves from each tooth. The tooth halves obtained were then individually stored in beakers (Multi-purpose containers, Greiner Bio-One, Frickenhausen, Germany), filled with PBS solution and refrigerated at 5 °C for further processing.

### Excavation

In order to minimize individual errors in dentin caries excavation, five experienced dentists participated in this examination, which was previously calibrated. For this purpose, they had to excavate an additional 10 teeth with the PolyBur P1 bur for training purposes prior to the start of the main study. Previously, the concept of caries excavation with polymer burs was explained to the dentists by one of the authors (TD) and the procedure according to the manufacturer’s recommendations was explained in detail. For the excavation of the dentin caries, the polymer bur was used in a slow-running handpiece at 2,000 rpm to 8,000 rpm and with light, discrete strokes leading out from the center of the lesion [21]. The speed of rotation was precisely controlled on the digital display of the dental unit. The assessment of caries freedom corresponded to the procedure in the main study. The results of this training excavation were clinically evaluated by one of the authors (TD) and discussed with the dentists.

The 100 samples (= 50 teeth) were randomly divided into five groups with 10 teeth per group and distributed to the dentists. Thus, each dentist received 10 corresponding tooth halves stored in PBS solution, any number of PolyBur P1 burs per sample (ISO sizes 014, 018, 023), and new conventional tungsten carbide bud burs (H1 SE) with the same sizes. During the experiment, the number of PolyBur P1 burs used was unlimited. The excavation was repeated with each kind of bur until the dentist estimated the dentin to be caries-free or no more caries could be removed. The hardness on probing (dental probe DA 410R; Aesculap, Tuttlingen, Germany) was used as a parameter to assess the caries-free status of the dentin. The time required for excavation was measured by means of a stopwatch. Afterwards, the samples were again stored again in PBS solution.

The samples were then blinded, i.e., a dentist, who was not involved in the examination, numbered the samples at random. Thus, the examiner no longer knew which sample was excavated with which bur and which dentist carried out the treatment (single blind study).

### Sectioning and staining

After excavation, all teeth were embedded in a mold, using epoxy resin (Technovit, Heraeus Kulzer, Hanau, Germany) for histological examination. Longitudinal serial sections 200 μm thick were obtained from each sample by cutting throughout the complete extent of the cavity with a diamond saw (Modell 1600, Leitz, Wetzlar, Germany) parallel to the first sectional plane, which was obtained by splitting the tooth into two halves. As many sections as possible were produced to cover the complete cavity as well as the adjacent dentin for examination; whereby, the number of the sections obtained (between 5 and 12) depended on the size of the cavity. Every section was pasted on an object plate (Engelbrecht Medizin- und Labortechnik, Edermünde, Germany). The sections were then stained with Mallory-Azan. All teeth were stained separately. The sections were first stained for 15 min with acid fuchsine (0.25%). Subsequently, after removing surplus with filter paper, a second staining with 2C 084 Anilinblau-Orange Mallory (0.5 g Aniline blue, 2 g orange, 1 g phosphowolfram acid in 100 ml distilled water; Chroma-Gesellschaft, Münster, Germany) was performed. Finally, the sections were rinsed with ethanol (90%). Histologically, cariously altered collagen was stained red and healthy collagen dark blue [26].

### Microscopic evaluation

The microscopic examination was carried out by dichotomous evaluation (cariously altered or healthy collagen). By light microscopy (Wild Photomakroskop M 400, Leica, Bensheim, Germany) at 32-fold magnification it was decided whether – according to the histological staining – the collagen was cariously altered or not. The examiner was unaware of how the specimens were treated. After verification of the magnification factor via a stage micrometer (Bresser, Meade Instruments Europe, Rhede, Germany) the thickness of the altered collagen was measured (less or more than 1 mm).

### Statistical evaluation

The time recorded for caries excavation was analyzed using the paired t-test.

The percentage of teeth and sections with cariously altered or healthy collagen was determined in order to evaluate the efficiency of the different burs. The result was statistically evaluated using the non-parametric Wilcoxon test for combined random samples. The percentage of teeth and sections with a cariously altered collagen thicker than 1 mm in each specimen (tooth) was evaluated in the same manner.

A binary logistical regression was performed to determine the influence of the three variables (type of bur, tooth, and dentist) on the results of the statistical evaluation concerning the criteria “cariously altered collagen” or “cariously altered collagen thickness (> 1 mm)”.

## Results

### Time recorded for removing cariously altered collagen

On average, it took 254 (± 148) seconds to remove cariously altered collagen out of a split tooth with PolyBur P1 and 202 (± 129) seconds with the bud bur H1 SE, respectively. The difference was statistically not significant (*p* > 0.05).

### Evaluation of the caries excavation at tooth level

In the 50 tooth halves excavated with PolyBur P1, cariously altered collagen was found in 48 specimens (96%), whereas sound collagen was found in only 2 tooth halves (4%). Of the 50 tooth halves excavated with H1 SE, 42 had cariously altered collagen (84%) and 8 showed sound collagen (16%). This difference between the burs was statistically significant (*p* < 0.05).

Of the 48 tooth halves of the group PolyBur P1 in which cariously altered collagen remained, 39 had a layer thickness > 1 mm (81.25%) and 9 had a layer thickness < 1 mm (18.75%). In group H1 SE, the layer thickness of the cariously altered collagen was > 1 mm in 29 samples (69%) and < 1 mm in 13 samples (31%). This difference was also statistically significant (*p* < 0.05).

### Evaluation of the caries excavation at section level

The overall number of samples in the group bud bur H1 SE was 282 sections, and 289 sections in the group PolyBur P1, counting a total of 571 sections. In the group H1 SE an average of 5.64 (± 2.45) sections was obtained per tooth whereas in the group PolyBur P1 there were 5.78 (± 2.53) sections. Thus, there was no significant difference between the numbers of sections obtained (*p* > 0.05).

Out of the 571 sections, 344 had remaining cariously altered collagen (60.2%) and 227 sections showed sound collagen (39.8%). 289 sections were obtained and stained from the PolyBur P1 treated teeth. Here, in 66.1% (191 out of 289) of the sections cariously altered collagen remained, whereas 33.9% (98 out of 289) showed sound collagen. In the group bud bur H1 SE 282 sections were evaluated: 45.7% (129 out of 282) had remaining cariously altered collagen and 54.3% (153 out of 282) showed sound collagen. The difference between PolyBur P1 and tungsten carbide bud bur H1 SE was statistically significant (*p* = 0.004).

Overall, the layer of remaining cariously altered collagen was thicker than 1 mm in 50.3% of the sections (173 out of the 344). The remaining cariously altered collagen after PolyBur P1 excavation in 56% (107 out of 191) of the sections the layer of cariously altered collagen was thicker than 1 mm and in 44% (84 out of 191) thinner than 1 mm. In the H1 SE bud bur group in 43.1% (66 out of 153) of the sections the layer of cariously altered collagen was thicker than 1 mm and in 56.9% (87 out of 153) of the sections thinner than 1 mm. This difference was statistically significant (*p* < 0.05).

### Logistical regression

In order to identify the influencing factors (type of bur, tooth, and dentist) that had an effect on the remaining cariously altered collagen, a logistic regression was calculated.

As a dependent variable, it was considered whether cariously altered collagen had occurred or not (dichotomous classification of tooth section). Calculated over all 571 sections, the regression model was found to be significant (*p* < 0.001). The explanatory variable “type of bur” within the model had an explanatory value for the presence of cariously altered collagen at the sections (*p* = 0.003), while the variables “tooth” and “dentist” had no statistically significant influence (*p* > 0.05).

## Discussion

The purpose of this in vitro study was to compare the efficiency of a polymer bur (PolyBur P1) with conventional tungsten carbide bud burs (H1 SE) in a split tooth model. It must be emphasized, that it was not the aim of this investigation to assess the concept of minimal-invasive caries excavation with polymer burs. Rather, the purpose was to simply evaluate if sufficient amounts of cariously altered collagen in carious dentin can be removed by the aid of this polymer bur, because it is generally accepted that all soft, carious dentin should be removed prior to restoration [1].

The sample teeth, extracted for various reasons, were stored in PBS solution in a refrigerator. PBS solution does not alter carious dentin. Other storage media like alcohol, formalin or glutaraldehyde may react with the dentin collagen structure and might lead to a hardening of the dentin caries. In contrast, PBS solution possesses a slight antibacterial effect without changing the collagen structure of the dentin or denatured proteins [Ericson D., 2003, personal communication].

Excavating corresponding cavity halves minimize differences on the excavation results due to different extension, depth, localization, structure etc. of the caries. Five trained dentists performed the excavation in order to minimize individual mistakes during bur handling and/or dentin probing. The histological evaluation was performed by one single examiner who evaluated the histological sections without knowing how the specimens were treated.

Clinically, the exact end-point of caries excavation cannot be defined easily and continues to be subjective. Hence, caries diagnosis just by clinical, tactile and/or visual criteria such as surface hardness and dentin color or caries detector dyes are not completely reliable and thus can be used in a scientific examination only with restrictions because an individual dentist might be influenced by subjective experience [1, 2, 15, 17–19, 25]. Therefore, a histological examination is the ideal method of visualizing the carious changes in the dentin and to obtain reproducible results to evaluate excavation procedures [16, 21, 25, 27].

Regarding histological staining, it has to be noted that dentin caries can be divided into distinct layers. The outer layer is contaminated by bacteria causing a non-remineralizable necrotic collagen matrix. In contrast, the dentin of the inner layer contains sound, intact, un-denatured collagen fibers, retaining the cross-banded ultrastructure. Bacteria are much less frequently observed and, if the acid challenge is removed, the inner layer has the potential to remineralize [28–30]. Mallory-Azan staining allows differentiation between these two layers [31]. Healthy, non-altered collagen will be stained blue; whereas affected carious dentin will appear red [26]. From a histological point of view, it can therefore be assumed with a high probability that the cariously altered collagen stained red in this study corresponds clinically to caries, while the healthy collagen stained blue can be described clinically as caries-free dentin. In conclusion, the Mallory-Azan staining is a quite large-scale method which allows exact results [31]. This reliable method has been used in other similar studies [13, 27, 32–34]. However, it should be noted that the histological staining method used here can only approximate the limit of healthy or infected dentin. It is also important to note the cutting losses caused by the diamond saw, which can lead to false positive or false negative results if a carious lesion or a healthy layer is lost in the cut.

To the best of the authors’ knowledge, there is only one study available to date, assessing the effectiveness of PolyBur P1 in dentin caries excavation in vitro [21]. However, in that study PolyBur P1 was not compared with a conventional carbide bud bur (as done in the present study), but with another polymer bur (SmartBurs II; SS White, Lakewood, NJ, USA). In that study using polarized light microscopy, both polymer burs were able to remove caries in 80% of the samples and were thus equally effective. Nevertheless, data comparing caries excavation with PolyBur P1 with conventional bud burs is still missing.

Within the limitation of this in vitro study PolyBur P1 was significantly less effective in removing cariously altered collagen than the conventional tungsten carbide bud bur H1 SE. Only 33.9% of the PolyBur P1 treated specimens were histologically free from cariously altered collagen, whereas resulting 45.7% with the bud bur H1 SE. Furthermore, if cariously altered collagen remained, this layer was significantly more often thicker than 1 mm in the PolyBur P1 group than in the H1 SE group.

Interestingly to note that even after excavation with conventional bud burs less than 50% of the specimens were determined to be free of cariously altered collagen. Therefore, one can speculate about whether the danger of routine over-excavation with conventional carbide bud burs really is a clinical problem, as it is often discussed [1, 2, 17, 18, 22, 35]. However, the results of this study are in accordance with other those of similar studies [13, 27, 32]. One reason may be that dentists had difficulties in handling the small sections of teeth. Although, in a more clinical experiment, both bur systems might show better results concerning caries excavation but it can be assumed that the relation of caries-free specimens between PolyBur P1 and bud bur will be similar, because the difference was statistically highly significant.

As until today there is no other data available evaluating PolyBur P1 in comparison to conventional carbide bud burs, the data of the present study cannot be compared with other publications. However, since Usha and Ranjani [21] stated that there was no significant difference in effectiveness between PolyBur P1 and another polymer bur (SmartBurs II), the results of the present study are compared with results from studies in which other polymer burs (namely SmartPrep, SmartBur, and SmartBurs II) were compared to conventional stainless steel bud burs.

Using confocal laser scanning microscopy [2], micro-CT and digital imaging [19] as well as Mallory-Azan-staining [13], polymer burs were not able to remove all carious dentin and left significantly more dentin caries than conventional bud burs. Due to incomplete dentin caries excavation dentin surfaces excavated with polymer bur exhibited significantly lower bond strengths to dentin adhesives and composite resins than dentin surfaces excavated with conventional bud burs [12, 17]. In microbiologically in vitro evaluations, conventional bud burs produced a significantly greater reduction in the count of viable bacteria (*Streptococcus mutans* and *Lactobacilli*) compared to polymer burs [24]. Thus, significantly more bacterial remnants were histologically present after excavation with polymer burs [16]. Likewise in vivo, a significantly higher reduction in the bacterial count of *Streptococcus mutans* (colony forming units) was found after excavation with conventional bud bur compared with polymer bur (87.25% vs. 58.05% reduction) [36]. Hence, conventional bud burs were more effective than polymer burs in dentin caries excavation [2, 12–14, 16, 17, 19, 24, 36]. Clinically, polymer burs were able to remove dentin caries completely in 65% of the cases and carbon steel bur in 100%. However, this difference had no impact on the clinical outcome after 6 months [14].

On the other hand, it has also been described that histologically, there was no difference in dentin caries excavation between polymer burs and conventional bud burs [23, 25]. Also microbiologically, polymer burs were as effective as conventional carbide bud bur at removing infected carious dentin [15]. Polymer burs attained higher preservation of carious-affected dentin than carbide burs [18], because polymer rotary instruments removed significantly less healthy, non-carious dentin than carbide bud burs [22].

Nevertheless, it can be concluded that in the vast majority of studies, using different methods for evaluation, polymer burs were not able to remove a sufficient amount of carious dentin, which is in accordance with the results of the present study.

Furthermore, most studies showed that caries excavation with polymer burs was significantly more time consuming than with conventional carbide bud burs [2, 14, 16]. However, in other studies no significant difference in excavation time was determined [13, 23, 25], which is also in accordance with the results of the present study.

The null hypothesis tested in this study had to be rejected: tungsten carbide bud burs (H1 SP) were significantly more effective in removing cariously altered collagen during dentin caries excavation than the polymer bur PolyBur P1. If dentin caries remained, the layer of cariously altered collagen was significantly more often thicker than 1 mm in the polymer bur group than in the conventional bud bur group.

The hardness and/or the shape of the PolyBur P1 might not be adequate to remove infected dentin completely. Hence, the present findings indicate that this polymer bur should be modified in design and hardness, in order to ensure complete removal of cariously altered collagen. It should be noted that remaining bacteria in the dentin carry the risk of caries recurrence and always forms a reservoir of toxins even after the death of the microorganisms, whereby inflammatory processes in the pulp may be maintained [37].

## Conclusion

Within the limitations of this in vitro study it can be concluded that the polymer bur PolyBur P1 is significantly less effective in removing sufficient amounts of cariously altered collagen in dentin caries excavation than conventional bud burs.

## Data Availability

The dataset used and/or analyzed during the current study are available from the corresponding author on reasonable request.

## Abbreviations

H1: Tungsten carbide bur H1 SE
KHN: Knoop Hardness
P1: PolyBur P1
PBS: Phosphate-buffered saline solution
PEKK: Polyether-ketone-ketone
